# Macrophage-derived Fgl2 dampens antitumor immunity through regulation of Fc**γ**RIIB^+^CD8^+^ T cells in melanoma

**DOI:** 10.1172/jci.insight.182563

**Published:** 2025-03-24

**Authors:** Kelsey B. Bennion, Julia Miranda R.Bazzano, Danya Liu, Maylene Wagener, Chrystal M. Paulos, Mandy L. Ford

**Affiliations:** 1 Cancer Biology PhD program,; 2 Department of Surgery,; 3 Winship Cancer Institute,; 4 Immunology and Molecular Pathogenesis PhD program, and; 5 Emory Transplant Center, Emory University School of Medicine, Atlanta, Georgia, USA.

**Keywords:** Immunology, Oncology, Cancer immunotherapy, Macrophages, T cells

## Abstract

Cancer immunotherapy has emerged as a promising therapeutic modality but heterogeneity in patient responsiveness remains. Thus, greater understanding of the immunologic factors that dictate response to immunotherapy is critical to improve patient outcomes. Here, we show that fibrinogen-like protein 2 (Fgl2) is elevated in the setting of melanoma in humans and mice and plays a functional role in inhibiting the CD8^+^ T cell response. Surprisingly, the tumor itself is not the major cellular source of Fgl2. Instead, we found that macrophage-secreted Fgl2 dampens the CD8^+^ T cell response through binding and apoptosis of FcγRIIB^+^CD8^+^ T cells. This regulation was CD8^+^ T cell autonomous and not via an antigen-presenting cell intermediary, as absence of *Fcgr2b* from the CD8^+^ T cells rendered T cells insensitive to Fgl2 regulation. Fgl2 is robustly expressed by macrophages in 10 cancer types in humans and in 6 syngeneic tumor models in mice, underscoring the clinical relevance of Fgl2 as a therapeutic target to promote T cell activity and improve patient immunotherapeutic response.

## Abstract 2

This manuscript explores a new pathway between two cell types that could be manipulated to promote T cell activity and improve cancer patient response to immunotherapies.

## Introduction

The success of cancer immunotherapies depends on the quality and magnitude of the patient CD8^+^ T cell response at the tumor. The myeloid compartment of the immune system has become increasingly appreciated for its role in thwarting the T cell antitumor response (1–5). In addition to chronic antigen exposure and hypoxia at the tumor, receptor-ligand interactions between myeloid cells and CD8^+^ T cells can promote an environment where impaired T cell function, termed T cell exhaustion (6–9), limits patient immunotherapeutic response (10–12). For many cancer types, including melanoma, increased presence of the myeloid compartment at the tumor is correlated with poorer patient prognosis (5, 13, 14). Current FDA-approved therapies targeting receptor-ligand pairs such as PD-1/PD-L1 have shown immense clinical promise, but patient response rates are variable, ranging from 20%–60% in melanoma (15–18), and disease progression remains an obstacle. As such, identifying and targeting novel pathways to enhance the size and functionality of the patient’s tumor-specific CD8^+^ T cell response remains an important goal.

We and others have found that the inhibitory Fcγ receptor FcγRIIB is expressed on mouse and human CD8^+^ T cells, a finding that challenges the decades-held dogma that CD8^+^ T cells do not express the inhibitory receptor FcγRIIB (19–24). Furthermore, we found that FcγRIIB is upregulated on high-quality CD8^+^ tumor-infiltrating lymphocytes in patients with melanoma (23). FcγRIIB^+^CD8^+^ T cells possess higher proliferative ability and secrete more proinflammatory cytokines than their FcγRIIB^–^ counterparts (20, 23), making them imperative to the antitumor response. Importantly, FcγRIIB functions in a cell-autonomous manner to temper the CD8^+^ T cell response in the context of cancer, viral infection, and transplantation (20, 22, 23) and may limit patient response to immunotherapies such as immune checkpoint blockade (23).

We also discovered that fibrinogen-like protein 2 (Fgl2) may serve as a ligand of FcγRIIB on CD8^+^ T cells, insofar as soluble Fgl2 (sFgl2) mediates apoptosis of FcγRIIB^+^ CD8^+^ in vitro (20, 24). Fgl2 can act as a prothrombinase in its membrane-bound form and contributes to hypercoagulation (25–27). The secreted form of Fgl2, sFgl2, is the focal point of this work and acts as an immunosuppressive cytokine. Elevated systemic levels of Fgl2 have been observed in the settings of hepatitis, renal injury, endometriosis, and certain cancer types (28–32). Upregulation of Fgl2 by tumor and stromal cells (e.g., hepatocellular carcinoma and glioma) have been correlated with poor patient survival (27, 33–38). In mouse models of glioma and hepatocellular carcinoma, Fgl2 from the tumor conferred cancer cells with stem-like qualities (36), inhibited CD103^+^ dendritic cell maturation (32, 37), and skewed myeloid cells to a protumorigenic state (35, 39). Other immunosuppressive cell types such as regulatory T cells (Tregs) and prorepair macrophages have been shown to produce sFgl2 in nonmelanoma models (31, 40–42). In those studies, sFgl2 suppressed T cell function indirectly through inhibiting antigen-presenting cell (APC) maturation and antigen presentation. Thus, the therapeutic efficacy of using Fgl2-blocking antibodies has recently been explored in the context of viral infection and cancer (39, 43–45). Therein, the authors posited that Fgl2-blocking antibodies provide enhanced T cell infiltration through indirect regulation of T cells, by targeting the functionality of innate cells, tumor cells, or APCs. Dissimilarly, the work presented herein provides another potential mechanism for the enhanced efficacy of these antibodies: a regulatory pathway directly targeting CD8^+^ T cells independently of another cell such as an APC by signaling via FcγRIIB expressed on the CD8^+^ T cell itself.

Fgl2 has been shown by others to bind FcγRIIB on myeloid cells, dendritic cells, and B cells to negatively regulate cellular activation, effector function, and/or survival (39, 46). Work herein shows that Fgl2 secreted by macrophages is a consequential binding partner of FcγRIIB on CD8^+^ T cells and functions during melanoma challenge to decrease tumor control through reducing the pool of tumor-specific CD8^+^ T cells. Given that FcγRIIB marks a high-quality effector-like memory CD8^+^ T cell (20, 22, 23), the apoptosis and elimination of FcγRIIB^+^ CD8^+^ T cells has functional relevance in antitumor immunity. In this work, Fgl2 was elevated systemically upon tumor challenge but was most present at the tumor, shedding light on both a local and systemic mechanism of immunosuppression that macrophages can employ. Here, we present a receptor-ligand interaction between a cell-extrinsic factor, Fgl2, and a CD8^+^ T cell–intrinsic factor, FcγRIIB, that ultimately negatively regulate the T cell antitumor response. These data support the protumor role of macrophage-derived Fgl2 in dysregulating the T cell antitumor response through CD8^+^ T cell–expressed FcγRIIB.

## Results

### Fgl2 is elevated in melanoma and is immunosuppressive to tumor-specific CD8^+^ T cells.

**Figure 1:**
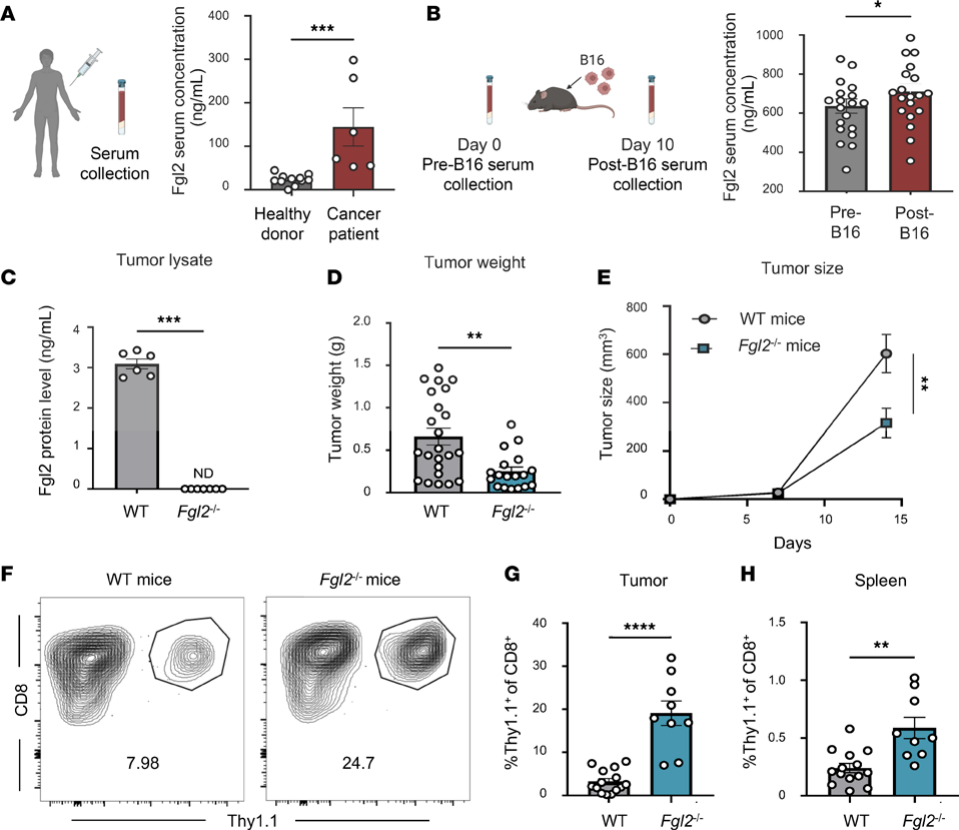
Fgl2 is elevated in melanoma and is immunosuppressive to tumor-specific CD8^+^ T cells. (**A**) Diagram and summary data of Fgl2 protein concentration through ELISA from the serum of healthy donors (*n* = 11) and patients with melanoma (*n* = 6). (**B**) Diagram and summary data of Fgl2 protein concentration through ELISA from the serum of mice before (pre-B16) and after challenge (post-B16) with B16-F10 melanoma (*n* = 20, pooled data from 2 experiments). (**C**) Summary data showing Fgl2 protein level in the tumor lysate of WT vs. *Fgl2*^–/–^ mice (*n* = 7–8, pooled data from 2 experiments). (**D**) Summary data showing tumor weight of WT vs. *Fgl2*^–/–^ mice given WT OT-I 14 days after tumor challenge (*n* = 18–23, pooled data from 3 experiments) and (**E**) a tumor growth curve measuring tumor size across a 14-day period (*n* = 10, pooled data from 3 experiments). (**F**) Summary data showing frequency of OT-I (Thy1.1^+^) at the (**G**) tumor and the (**H**) spleen in WT vs. *Fgl2*^–/–^ mice 14 days after tumor challenge (*n* = 9–14, pooled data from 2 experiments). Mann-Whitney nonparametric, unpaired *t* test was used when comparing 2 groups. Wilcoxon’s matched-pairs nonparametric test was used for normalized data. Summary data are presented as mean ± SEM. *P < 0.05; **P < 0.01; ***P < 0.001; ****P < 0.0001.

To begin to assess the role of sFgl2 in melanoma, sFgl2 was measured in the serum of human healthy donors and patients with melanoma. Compared with healthy donors, sFgl2 was significantly increased in the serum of patients with melanoma (Figure 1A). In a mouse model of melanoma, sFgl2 was similarly elevated in the serum of melanoma-bearing mice compared with pretumor challenge (Figure 1B). As the role of tumor-derived Fgl2 has been described, we sought to determine whether the tumor was a major cellular source of Fgl2 in our model. Fgl2 ELISA was first performed on B16 cells in culture and no detectable Fgl2 protein was found (data not shown). To interrogate the contribution of Fgl2 from the tumor versus tumor-associated cells that originate from the host in vivo, the tumors of WT or *Fgl2*^–/–^ mice were homogenized to prepare tumor lysate. In tumors from WT hosts, Fgl2 was detected in the tumor lysate, whereas Fgl2 was undetectable in the tumor lysate from *Fgl2*^–/–^ mice (Figure 1C), suggesting that B16 cancer cells are not a cellular source of Fgl2. Instead, cells originating from the host present at the tumor but not the tumor itself are predominant sources of Fgl2. We then interrogated the impact of Fgl2 from the host-derived Fgl2 by challenging WT or *Fgl2*^–/–^ recipients of WT T cell receptor–transgenic (TCR-transgenic) OVA-specific Thy1.1^+^ OT-I T cells with B16 OVA. Fgl2 deficiency resulted in significantly decreased tumor weight (Figure 1D) as well as tumor progression (Figure 1E). The decreased tumor size in *Fgl2*^–/–^ mice compared with WT mice was commensurate with an increased frequency of tumor-specific OT-I CD8^+^ T cells at the tumor (Figure 1, F and G) and spleen (Figure 1H) on day 14 after tumor implantation. Using the gp100/pmel-17 B16 mouse model of melanoma where pmel-17 transgenic CD8^+^ T cells recognize the self-antigen gp100 on the murine melanoma cells, we found that the immunosuppressive effect of host Fgl2 was conserved. In addition to increased pmel-17 CD8^+^ infiltration into the tumor (Supplemental Figure 1, A–C; supplemental material available online with this article; https://doi.org/10.1172/jci.insight.182563DS1), *Fgl2*^–/–^ mice exhibited decreased tumor size and weight compared with WT B16 tumor–bearing mice (Supplemental Figure 1, D and E). To determine potential effector molecules impacting the enhanced fitness of tumor-specific CD8^+^ T cells in *Fgl2*-deficient mice, we next phenotypically and functionally characterized OT-I from healthy and tumor-bearing WT versus *Fgl2*^–/–^ mice. No differences were observed in T cell activation markers CD69, CD25, or CD44, or in TNF and IL-2 cytokine production in healthy mice (Supplemental Figure 2A). However, IFN-γ production by CD8^+^ T cells was enhanced in *Fgl2*^–/–^ mice compared with WT mice, an observation in line with the findings of several groups that Fgl2 can suppress all CD8^+^ T cells (36, 37, 47). This difference in IFN-γ production by OT-I upon ex vivo peptide stimulation was not maintained after tumor challenge. The frequency of PD-1^+^, CD44^hi^, Ki67^+^, TNF^+^, and IFN-γ^+^ CD8^+^ T cells at the tumor were also unchanged in comparing WT versus *Fgl2*^–/–^ mice (Supplemental Figure 2B). Given the observed role of Fgl2 from the host in suppressing antitumor efficacy, we next sought to identify the mechanism by which Fgl2 mediated this suppressive effect.

### Regulation of FcγRIIB^+^CD8^+^ T cells is dependent on host Fgl2 and not tumor-derived Fgl2.

**Figure 2:**
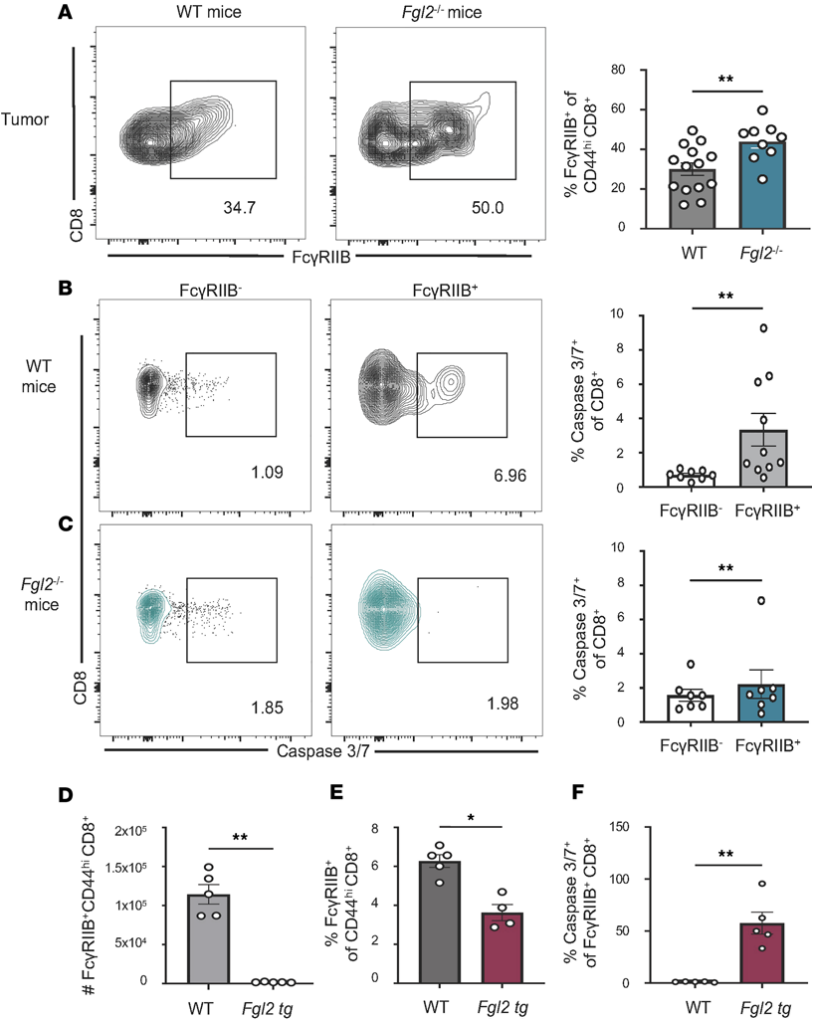
Regulation of FcγRIIB^+^CD8^+^ T cells is dependent on host Fgl2 and not tumor-derived Fgl2. Representative flow cytometry plots and summary data of frequency of FcγRIIB^+^ among CD44^hi^CD8^+^ T cells in the (**A**) tumor of WT vs. *Fgl2*^–/–^ B16-challenged mice (*n* = 9–14, pooled data from 2 experiments). Representative flow plots and summary data showing the frequency of caspase 3/7^+^FcγRIIB^–^ vs. caspase 3/7^+^FcγRIIB^+^ CD8^+^ T cells in (**B**) WT vs. (**C**) *Fgl2*^–/–^ tumor-bearing mice (*n* = 7–10, pooled data from 2 experiments). On day 14, WT or Fgl2-overexpressing transgenic mice were harvested for analysis of spleens. Summary data showing the (**D**) cell count and (**E**) frequency of FcγRIIB^+^CD44^hi^CD8^+^ T cells in WT vs. Fgl2-Tg B16-bearing mice. (**F**) Summary data showing frequency of caspase 3/7^+^ cells among FcγRIIB^+^CD8^+^ cells in WT vs. Fgl2-transgenic B16-bearing mice (*n* = 5). Mann-Whitney nonparametric, unpaired test was used when comparing 2 groups. Summary data are presented as mean ± SEM. *P < 0.05; **P < 0.01.

Our previous studies showed that sFgl2 may serve as a binding partner for FcγRIIB on CD8^+^ T cells, as incubation with sFgl2 induced apoptosis of FcγRIIB^+^CD8^+^ cells in vitro (20, 24). Because FcγRIIB is expressed on tumor-specific CD8^+^ T cells (20–23), we posited that sFgl2 may temper the T cell antitumor response by binding FcγRIIB^+^CD8^+^ cells to induce apoptosis of FcγRIIB^+^CD8^+^ T cells in vivo. First, we quantified the frequency of FcγRIIB^+^ cells among CD44^hi^CD8^+^ T cells at the draining lymph node, spleen, and tumor of WT mice (Supplemental Figure 3, A and B). We found that FcγRIIB is most expressed at the site of most antigen, the tumor. This result is in keeping with previous data from our lab showing that FcγRIIB is upregulated upon TCR stimulation. Next, we asked whether the absence of the ligand, Fgl2, would rescue FcγRIIB^+^CD8^+^ T cells in vivo. To test this hypothesis, WT or *Fgl2*^–/–^ mice were challenged with B16-OVA. Upon flow analyses of the draining lymph node (Supplemental Figure 3C), spleen (Supplemental Figure 3D), and tumor (Figure 2A) of these mice on day 14, we found significantly increased frequencies of FcγRIIB^+^ cells among CD44^hi^CD8^+^ T cells in *Fgl2*^–/–^ mice compared with WT mice. We next measured apoptosis via caspase 3/7 staining of FcγRIIB^+^CD8^+^ T cells versus FcγRIIB^–^ cells. We found that significantly more CD8^+^ T cells within the FcγRIIB^+^ subset underwent apoptosis compared with cells in the FcγRIIB^–^ compartment in WT tumor-bearing mice (Figure 2B). In *Fgl2*^–/–^ mice, FcγRIIB^+^ cells did not undergo more apoptosis compared with FcγRIIB^–^ T cells (Figure 2C). To establish a more linear relationship between apoptosis and the FcγRIIB/Fgl2 axis, transgenic mice that constitutively express Fgl2 received melanoma tumors and results demonstrated a significant reduction in the cell number (Figure 2D) and frequency (Figure 2E) of FcγRIIB^+^CD44^hi^CD8^+^ T cells in B16 tumor–bearing Fgl2-transgenic mice compared with WT mice. This reduction in the FcγRIIB^+^CD8^+^ T cell compartment was accompanied by significantly more FcγRIIB^+^CD8^+^ T cells undergoing apoptosis, as measured by caspase 3/7 staining in Fgl2-overexpressing mice compared with WT mice (Figure 2F). These experiments revealed that host-derived Fgl2 is sufficient to negatively regulate FcγRIIB^+^CD8^+^ T cells in vivo, and in turn, regulate the host CD8^+^ T cell response.

### Fgl2 selectively regulates WT but not Fcgr2b^–/–^ tumor-specific CD8^+^ T cells in vivo.

**Figure 3:**
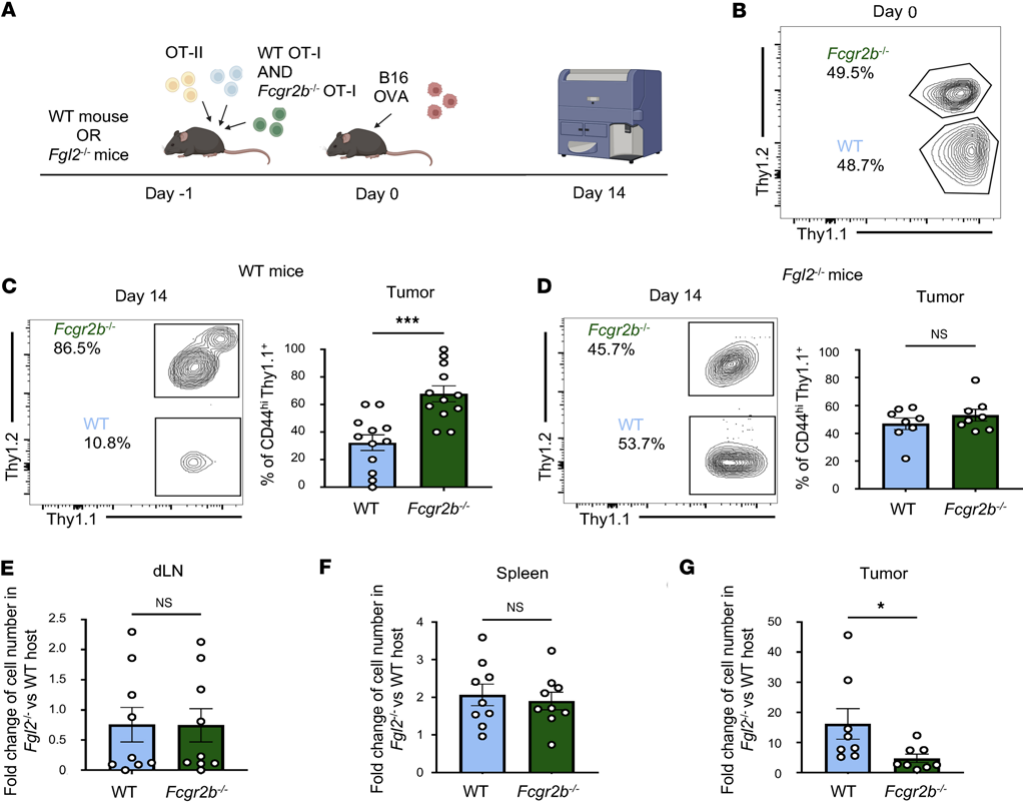
Fgl2 selectively regulates WT but not *Fcgr2b*^–/–^ tumor-specific CD8^+^ T cells in vivo. (**A**) Schematic wherein WT or *Fgl2*^–/–^ mice were given WT and *Fcgr2b*^–/–^ OT-I CD8^+^ T cells at a 1:1 ratio and OT-II CD4^+^ T cells 1 day prior to cancer cell inoculation. Fourteen days later, tumors were harvested to assess OT-I populations by flow cytometry. (**B**) Representative flow plot showing day 0 staining of WT (Thy1.1^+^) and *Fcgr2b*^–/–^ (Thy1.1^+^Thy1.2^+^) OT-I prior to co-adoptive transfer. Representative flow plot and summary data of WT (Thy1.1^+^) vs. *Fcgr2b*^–/–^ (Thy1.1^+^Thy1.2^+^) OT-I at the tumor of (**C**) WT (*n* = 12, pooled data from 2 experiments) vs. (**D**) *Fgl2*^–/–^ (*n* = 8, pooled data from 2 experiments) mice 14 days after tumor challenge. Summary data showing the fold change in cell number of each OT-I subset using the cell number of each OT-I subset in the *Fgl2*^–/–^ host relative to the WT host in the (**E**) draining lymph node (dLN), (**F**) spleen, and (**G**) tumor. Cells shown were gated on Thy1.1^+^CD8^+^ cells. Mann-Whitney nonparametric, unpaired *t* test was used when comparing 2 groups. Summary data are presented as mean ± SEM. *P < 0.05, ***P < 0.001.

In our previous studies, *Fcgr2b*^–/–^ OVA-specific CD8^+^ T cells outcompeted WT OVA-specific CD8^+^ T cells in a WT mouse in the context of skin graft and melanoma (20, 22). To understand whether this advantage occurred because *Fcgr2b*^–/–^ OT-I were insensitive to Fgl2-mediated apoptosis via FcγRIIB binding, we employed a model wherein *Fcgr2b*^–/–^ and WT OT-I CD8^+^ T cells were coadoptively transferred at a 1:1 ratio into mice that were then challenged with B16-OVA (Figure 3, A and B). Mice were sacrificed on day 14 and OT-I CD8^+^ T cells at the tumor were measured. As expected, in WT mice, *Fcgr2b*^–/–^ OT-I were present at a significantly higher frequency than WT OT-I CD8^+^ T cells (Figure 3C). However, in global *Fgl2*^–/–^ mice, the advantage of *Fcgr2b*^–/–^ OT-I over WT OT-I disappeared such that there was not a significant difference in frequency between OT-I subsets (Figure 3D). As an additional readout, the fold change in the cell number of WT and *Fcgr2b*^–/–^ OT-I in the *Fgl2*^–/–^ host was calculated using the cell numbers of each subset in the WT host as a baseline measure. Although more cells were present in both OT-I subsets in *Fgl2*^–/–^ mice compared with WT mice at all sites measured, there was not a significant increase in WT over *Fcgr2b*^–/–^ OT-I in the draining lymph node (Figure 3E) or spleen (Figure 3F) in *Fgl2*^–/–^ mice. However, WT OT-I were preferentially increased at the tumor compared with *Fcgr2b*^–/–^ OT-I (Figure 3G) in *Fgl2*^–/–^ hosts compared with WT hosts. Taken together, these data support the conclusion that regulation of FcγRIIB-competent tumor-specific CD8^+^ T cells, but not *Fcgr2b*^–/–^ CD8^+^ T cells, are uniquely regulated via Fgl2. These data also suggest that the regulation of FcγRIIB^+^CD8^+^ cells by Fgl2 at the tumor occurs in a CD8^+^ T cell–direct manner and not indirectly through an APC intermediary, as genetic deletion of FcγRIIB prevented regulation via Fgl2.

### Immune-derived Fgl2 negatively regulates FcγRIIB^+^CD8^+^ T cells through apoptosis.

**Figure 4:**
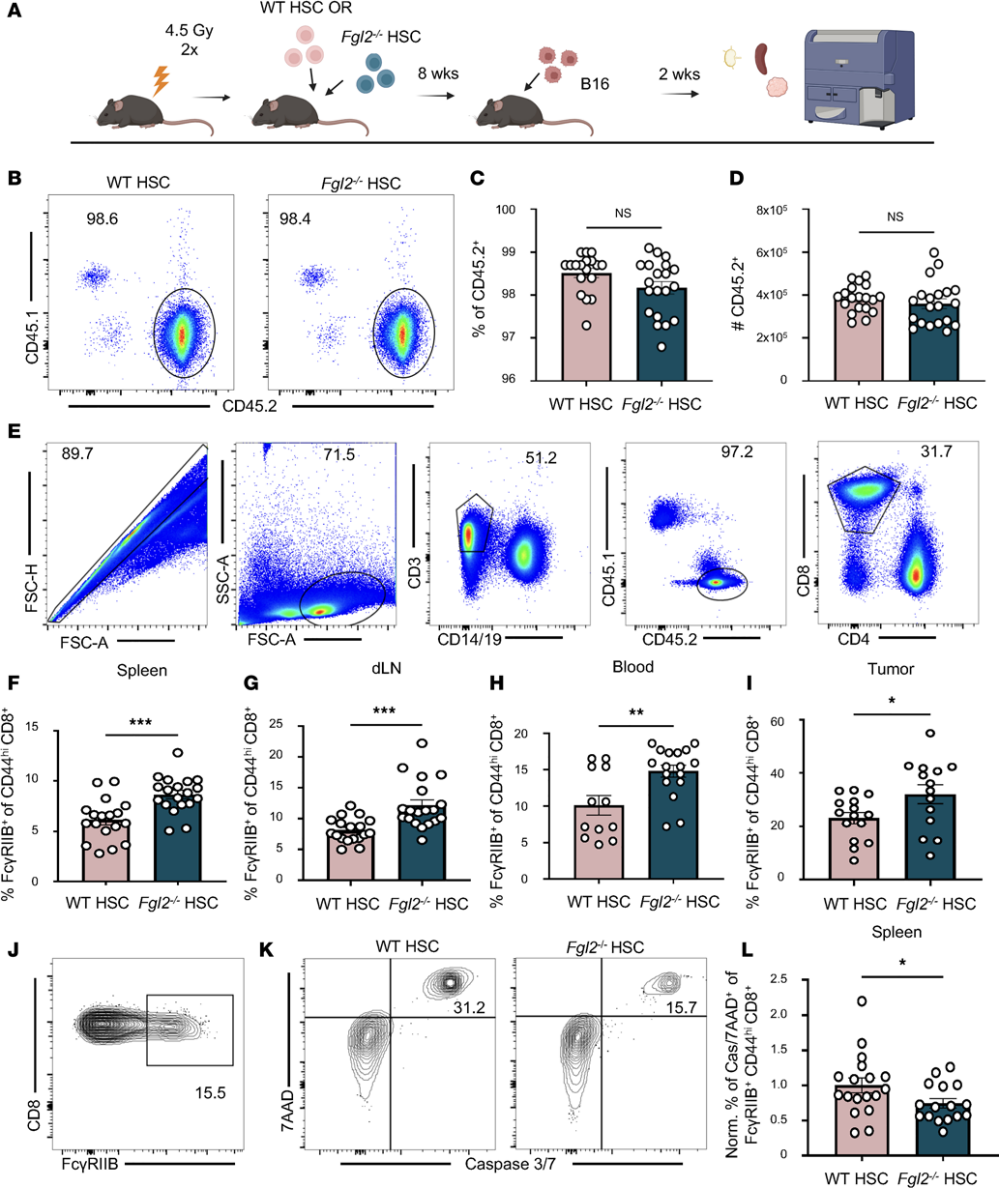
Immune-derived Fgl2 negatively regulates FcγRIIB^+^CD8^+^ T cells through apoptosis. (**A**) Schematic wherein CD45.1^+^ congenic mice were irradiated prior to reconstitution with CD45.2^+^ WT or *Fgl2*^–/–^ hematopoietic stem cells (HSCs). After 8 weeks, mice were injected with B16-F10. Fourteen days later, mice were sacrificed for harvest of blood, draining lymph node (dLN), spleen, and tumor. (**B**) Representative flow plots showing approximately 98% chimerism in mice reconstituted with CD45.2^+^ WT or *Fgl2*^–/–^ HSCs. Summary data showing the (**C**) frequency and (**D**) cell number of CD45.2^+^ cells from the blood 8 weeks after reconstituted with CD45.2^+^ WT or *Fgl2*^–/–^ HSCs (*n* = 19–20, pooled data from 3 experiments). (**E**) Gating strategy employed prior to FcγRIIB staining on CD8^+^ T cells. As FcγRIIB is known to be expressed on myeloid and B cells, the gating strategy employed serves to exclude contaminating CD14^+^ or CD19^+^ cells. Summary data showing FcγRIIB staining among CD44^hi^CD8^+^ T cells in the (**F**) spleen (*n* = 18–19, pooled data from 3 experiments), (**G**), dLN (*n* = 18–19, pooled data from 3 experiments), (**H**) blood (*n* = 8–10, pooled data from 2 experiments), and (**I**) tumor (*n* = 12–13, pooled data from 2 experiments) of mice reconstituted with WT vs. *Fgl2*^–/–^ HSCs. (**J**) Representative flow plot showing FcγRIIB staining prior to caspase 3/7 and 7AAD staining of FcγRIIB^+^CD44^hi^CD8^+^ cells in WT or *Fgl2*^–/–^ HSC–reconstituted mice. (**K**) Representative flow plots and (**L**) summary data showing the normalized frequency of caspase 3/7^+^7AAD^+^ within the FcγRIIB^+^CD44^hi^CD8^+^ T cell population in the spleens of WT HSC mice vs. caspase 3/7^+^7AAD^+^ within the FcγRIIB^+^CD44^hi^CD8^+^ T cell population in the spleens of *Fgl2*^–/–^ HSC–reconstituted mice (*n* = 12–14, pooled data from 2 experiments). The frequency of populations in *Fgl2*^–/–^ HSC–reconstituted mice was normalized to the average of WT HSC–reconstituted mice. Mann-Whitney nonparametric, unpaired *t* test was used when comparing 2 groups. Summary data are presented as mean ± SEM. *P < 0.05, **P < 0.01, ***P < 0.001.

We next sought to identify the cellular source of Fgl2 that regulates the T cell response through FcγRIIB. As Fgl2 has been shown to be produced by both hematopoietic and nonhematopoietic cell types by other groups, we generated bone marrow chimeras to determine the impact of immune cell–derived Fgl2 (Figure 4A). After sublethal irradiation, CD45.1 congenic mice were reconstituted with WT or *Fgl2*^–/–^ hematopoietic stem cells (HSCs). After confirmation of a similar immune cell reconstitution between WT HSC– and *Fgl2*^–/–^ HSC–reconstituted mice 8 weeks later (Figure 4, B–D), bone marrow chimeric mice were challenged with B16-F10 melanoma cells. Fourteen days later, the spleen, draining lymph node, blood, and tumor of mice were harvested for flow analyses (Figure 4E). Mice reconstituted with *Fgl2*^–/–^ HSCs exhibited increased frequencies of FcγRIIB^+^CD44^hi^CD8^+^ T cells compared with mice reconstituted with WT HSCs at all sites measured (Figure 4, F–I). This observed increase in FcγRIIB^+^CD44^hi^CD8^+^ T cells in *Fgl2*^–/–^ HSC–reconstituted mice challenged with B16-F10 was accompanied by a concomitant increase in apoptosis of FcγRIIB^+^CD44^hi^CD8^+^ T cells (Figure 4, J–L), as measured by caspase 3/7 and 7AAD flow staining. Further elucidating the detrimental impact of FcγRIIB^+^CD8^+^ T cell death, linear regression showed that the frequency of FcγRIIB^+^CD8^+^ T cells was negatively associated with tumor size in WT HSC–reconstituted mice (*R*^2^ = 0.58, *P* = 0.0101; Supplemental Figure 4A) but not in *Fgl2*^–/–^ HSC–reconstituted mice (Supplemental Figure 4B). These data support the role of a hematopoietic source of Fgl2 in regulating antigen-specific CD8^+^ T cell responses via apoptosis of FcγRIIB^+^ cells systemically and locally at the tumor site.

### Myeloid cells are a predominant cellular source of Fgl2 in humans with cancer.

**Figure 5:**
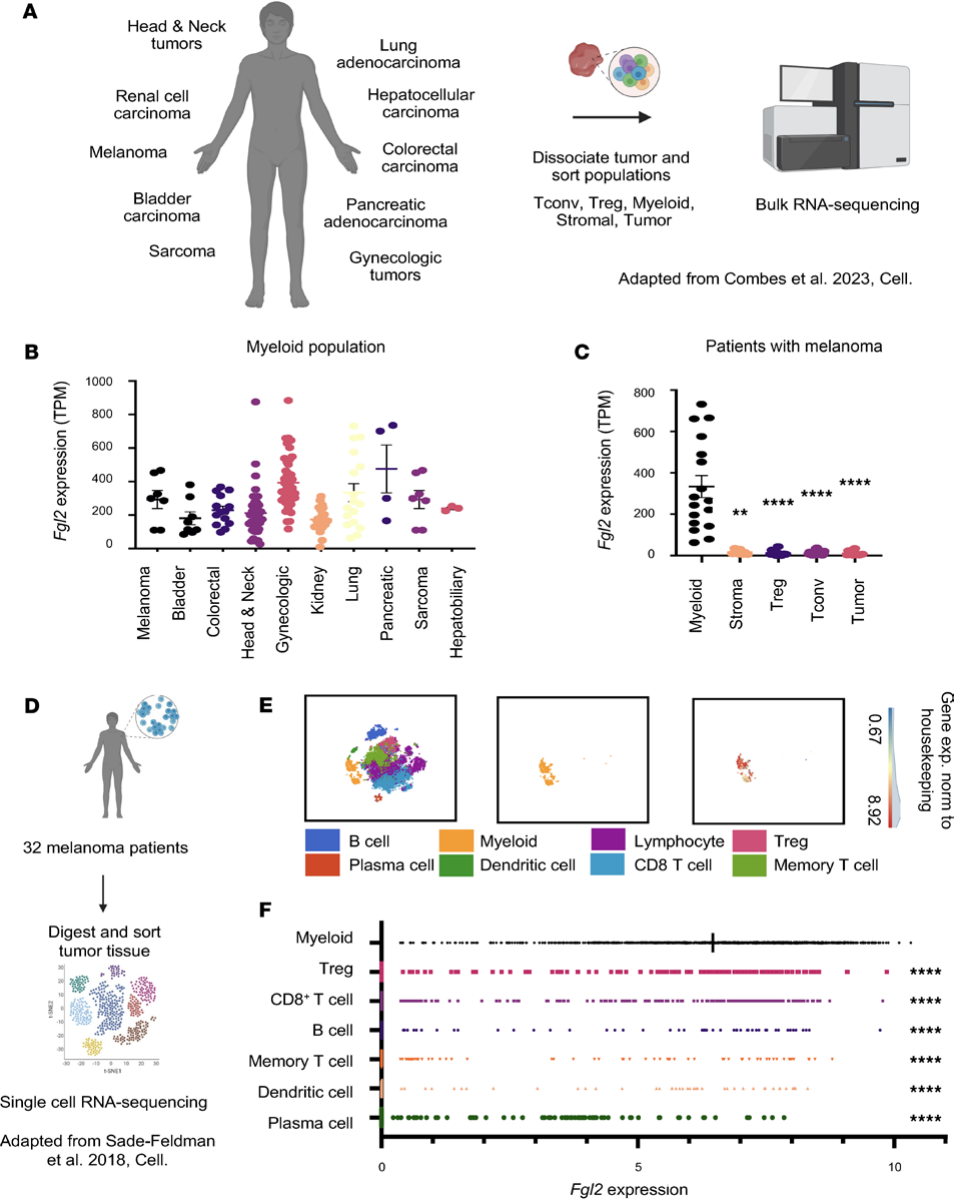
Myeloid cells are a predominant cellular source of Fgl2 in humans with cancer. (**A**) Schematic of the workflow previously published by Combes et al. (51) showing dissociation of tumors for bulk RNA sequencing from patients with head and neck tumors (*n* = 38), lung adenocarcinoma (*n* = 17), renal cell carcinoma (*n* = 21), hepatocellular carcinoma (*n* = 3), melanoma (*n* = 7), colorectal carcinoma (*n* = 14), bladder carcinoma (*n* = 8), pancreatic adenocarcinoma (*n* = 4), gynecologic tumors (*n* = 43), and sarcomas (*n* = 7). Cancer types were excluded if fewer than 3 samples were present in the myeloid population in the cancer type. (**B**) Dot plots showing the expression of *Fgl2* in the sorted myeloid population of patients across 10 different cancer types. (**C**) Dot plots showing the expression of *Fgl2* in the sorted myeloid (*n* = 17), stroma (*n* = 7), Treg (*n* = 13), Tconv (*n* = 21), and tumor (*n* = 12) populations in patients with melanoma. (**D**) Schematic of workflow published by Sade-Feldman et al. (52) of the publicly available dataset consisting of 16,291 single-cell transcriptome profiles from patient tumors (*n* = 32). (**E**) *t*-Distributed stochastic neighbor embedding (tSNE) plots showing *Fgl2* expression in the myeloid population at these patient tumors; data were normalized to housekeeping gene expression. (**F**) Accompanying bar graph for the tSNE plot showing *Fgl2* expression in the myeloid population as well as other tumor-infiltrating immune populations. Mann-Whitney non-parametric, unpaired *t* test was used when comparing 2 groups; Kruskall-Wallis nonparametric, 1-way ANOVA was used when comparing more than 2 groups. Summary data are presented as mean ± SEM. **P < 0.01, ****P < 0.0001.

To investigate the presence of Fgl2 in patients ith skin cutaneous melanoma (SKCM) more broadly, we queried the expression of Fgl2 in patients with primary melanoma versus melanoma metastasis through the tumor immune estimation resource (TIMER) database (48–50). Fgl2 expression was significantly increased in patients with SKCM metastasis compared with SKCM primary tumor (*P* = 0.458 × 10^–10^) (Supplemental Figure 4C). Furthermore, in patients with SKCM, Fgl2 expression was positively correlated with macrophage infiltration into the tumor (Supplemental Figure 4D). To investigate which immune cell subsets could be sources of Fgl2 in patients with cancer, we reanalyzed the publicly available dataset deposited by Combes et al. (51), wherein tumors from a myriad of cancer types were dissociated and populations of conventional T cells (Tconv), regulatory T cells (Tregs), myeloid cells, stromal cells, and tumor cells were sorted for bulk RNA sequencing (Figure 5A). Across 10 different cancer types, *Fgl2* was robustly expressed in the sorted myeloid population (Figure 5B). In patients with melanoma, myeloid cells were a substantial source of *Fgl2* compared with other sorted populations (Figure 5C); this finding was consistent across 7 other cancer types (Supplemental Figure 5, A–H). To validate these data at a single-cell level, we reanalyzed a second publicly available dataset from Sade-Feldman et al. (52), wherein single-cell RNA sequencing was performed on tumors from patients with melanoma (*n* = 32) (Figure 5D). Myeloid cells expressed significantly more *Fgl2* compared with several other immune cell types (Figure 5, E and F). Coupled together, these data allow us to conclude that across various cancer types, myeloid cells are a considerable source of *Fgl2* at the RNA level in humans.

### Macrophages from tumor-challenged mice secrete Fgl2 and most at the tumor.

**Figure 6:**
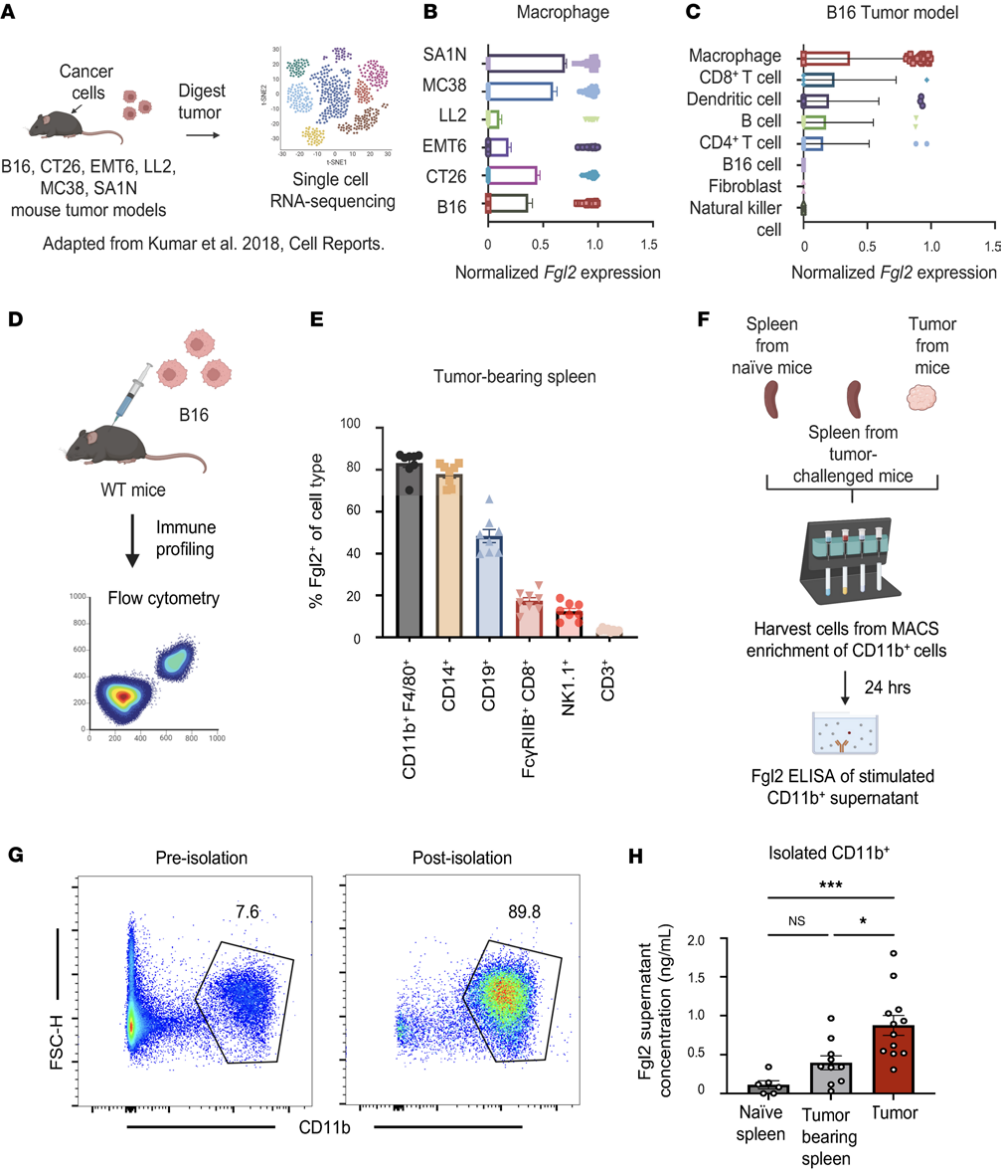
Macrophages from tumor-challenged mice secrete Fgl2 and most at the tumor. (**A**) Schematic showing the experimental design of a previously published study by Kumar et al. (74) where single-cell RNA sequencing was performed on the tumors from 6 syngeneic mouse tumor models. (**B**) Bar graphs showing expression of *Fgl2* (TPM normalized to housekeeping expression) on macrophages at the tumors in B16 (*n* = 133 cells), CT26 (*n* = 356 cells), EMT6 (*n* = 212 cells), LL2 (*n* = 167 cells), MC38 (*n* = 169 cells), and SA1N (*n* = 735 cells) mouse tumor models. (**C**) Bar graphs showing expression of *Fgl2* (TPM normalized to housekeeping gene) in tumor-infiltrating populations within the B16 model. (**D**) Diagram and summary data of immune profiling of populations at the (**E**) spleen (*n* = 8) and (**F**) tumor (*n* = 7) of B16-challenged mice. Representative data from 2 experiments are shown. (**G**) Representative flow showing the pre- and postisolation purity of enriched CD11b^+^ splenocytes from naive spleen, tumor-challenged spleen, or tumor. The supernatant of CD11b^+^ isolated cells was then collected after 24 hours for Fgl2 ELISA. (**H**) Summary data quantifying Fgl2 concentration in the supernatant of WT CD11b^+^ cells (*n* = 3–6 mice per group). Representative data from 2 experiments is shown. Kruskall-Wallis nonparametric, 1-way ANOVA was used when comparing more than 2 groups. Summary data are presented as mean ± SEM. *P < 0.05, ***P < 0.001.

As macrophages compose a large portion of tumor-infiltrating cells, we then asked whether macrophages could express Fgl2 at the tumor in mice. Through analysis of a publicly available dataset of 6 syngeneic mouse tumor models (Figure 6A), we found that macrophages expressed *Fgl2* in all mouse tumor models tested (Figure 6B), with appreciable *Fgl2* expression in the B16 model compared with other tumor-infiltrating populations (Figure 6C). To measure Fgl2 at the protein level in the B16 mouse model, we challenged WT mice with B16 cancer cells for downstream immune profiling (Figure 6D). After tumor challenge, mice were sacrificed and Fgl2 expression of several immune cell types was measured. We observed that the CD11b^+^F4/80^+^ macrophage population robustly expressed Fgl2 in the spleen of tumor-challenged mice (Figure 6E).

Because macrophages are known to express membrane-bound Fgl2 as well as sFgl2, the ability of macrophages to secrete sFgl2 (which binds to FcγRIIB) was assessed through isolation of CD11b^+^ cells from naive spleen, tumor-challenged spleen, or tumor of WT mice (Figure 6, F and G). The level of Fgl2 in the supernatant of these cells was then quantified through Fgl2 ELISA. sFgl2 was highest in the supernatant of CD11b^+^ cells isolated from the tumor of WT mice, followed by cells from the spleen of tumor-challenged mice, and lowest in CD11b^+^ cells isolated from the spleen of naive mice (Figure 6H). These data confirm that macrophages express Fgl2 across many types of murine tumors and that Fgl2 is most secreted by CD11b^+^ cells at the tumor of melanoma-bearing mice.

### Macrophage-secreted Fgl2 induces apoptosis of WT but not Fcgr2b^–/–^ tumor-specific CD8^+^T cells.

**Figure 7:**
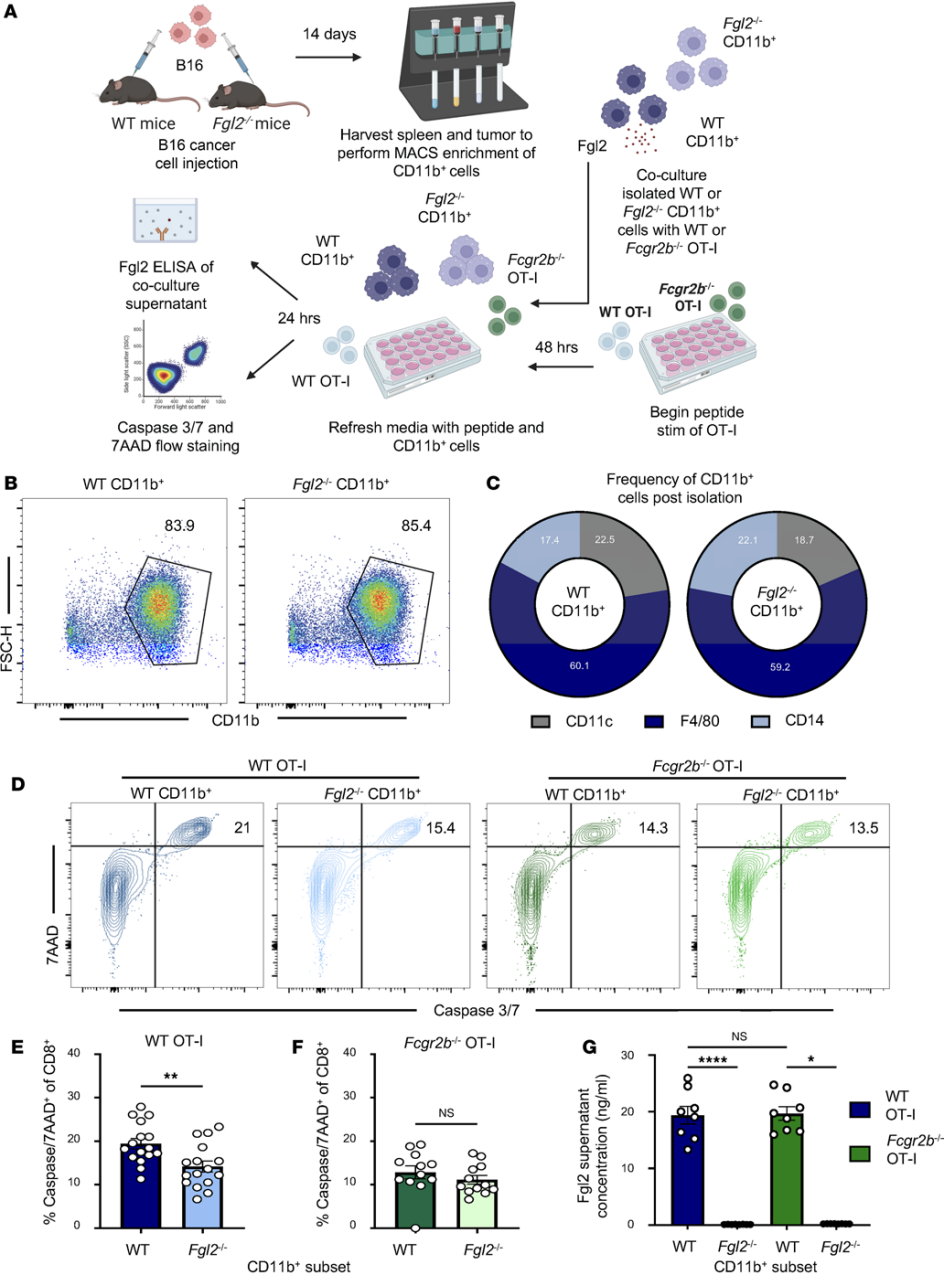
Macrophage-secreted Fgl2 induces apoptosis of WT but not *Fcgr2b^–/–^* tumor-specific CD8^+^ T cells. (**A**) Schematic showing that WT and *Fgl2*^–/–^ mice were challenged with B16 cells and 14 days later, CD11b^+^ splenocytes were MACS enriched with CD11b microbeads. Two days prior to CD11b^+^ cell isolation, splenocytes from WT or *Fcgr2b*^–/–^ OT-I transgenic mice were stimulated with OVA_257–264_ (SIINFEKL). Upon CD11b^+^ isolation as described above, 2-day-stimulated OT-I T cells were cocultured with WT or *Fgl2*^–/–^ CD11b^+^ cells. Twenty-four hours later, cell supernatant was collected for Fgl2 ELISA and OT-I T cells were collected for caspase 3/7 and 7AAD flow staining. (**B**) Representative flow plots showing similar frequency of CD11b^+^ cells isolated from WT or *Fgl2*^–/–^ mice. (**C**) Pie graphs showing the frequency of CD11c^+^, F4/80^+^, and CD14^+^ cells within the CD11b^+^ cell isolate (*n* = 5, average value is shown). (**D**) Representative flow plots showing caspase 3/7 and 7AAD staining of WT and *Fcgr2b*^–/–^ OT-I cocultured with WT or *Fgl2*^–/–^ CD11b^+^ cells. Bar graphs showing frequency of caspase 3/7^+^7AAD^+^ (**E**) WT (*n* = 16, pooled data from 2 experiments) vs. (**F**) *Fcgr2b*^–/–^ OT-I (*n* = 12, pooled data from 2 experiments) cocultured with WT or *Fgl2*^–/–^ CD11b^+^ cells. (**G**) Bar graph showing Fgl2 protein concentration of WT CD11b^+^ cells incubated with WT or *Fcgr2b*^–/–^ OT-I, with no detectable protein signal in the *Fgl2*^–/–^ CD11b^+^ cells incubated with WT or *Fcgr2b*^–/–^ OT-I (*n* = 8, representative data from 2 experiments). Mann-Whitney nonparametric, unpaired *t* test was used when comparing 2 groups; Kruskall-Wallis nonparametric, 1-way ANOVA was used when comparing more than 2 groups. Summary data are presented as mean ± SEM. *P < 0.05, **P < 0.01, ****P < 0.0001.

Lastly, we sought to determine whether Fgl2 secreted by CD11b^+^ isolated from tumor-challenged mice was sufficient to induce apoptosis of FcγRIIB-competent but not *Fcgr2b*^–/–^ OVA-specific CD8^+^ T cells. The experimental design from above was used to isolate CD11b^+^ cells from tumor-challenged WT or *Fgl2*^–/–^ mice. Two days prior, WT and *Fcgr2b*^–/–^ OT-I were stimulated with their cognate antigen. At the time of CD11b^+^ cell isolation, previously stimulated OT-I cells were cocultured with CD11b^+^ cells isolated from tumor-challenged mice for 24 hours (Figure 7, A and B). F4/80^+^ cells composed the largest proportion of isolated CD11b^+^ cells compared with CD11c^+^ and CD14^+^, demonstrating that the majority of isolated CD11b^+^ cells were macrophages (Figure 7C). After coculture, caspase 3/7 and 7AAD staining was performed on OT-I CD8^+^ T cells to determine the extent of apoptosis that occurred upon coculture with WT or *Fgl2*^–/–^ CD11b^+^ cells (Figure 7C). Critically, FcγRIIB-competent WT OT-I underwent significantly more apoptosis when stimulated in the presence of WT CD11b^+^ cells compared with WT OT-I stimulated in the presence of *Fgl2*^–/–^ CD11b^+^ cells (Figure 7, D and E). No difference in apoptosis was observed when *Fcgr2b*^–/–^ OT-I were cultured with WT CD11b^+^ cells compared to *Fgl2*^–/–^ CD11b^+^ cells (Figure 7, D and F). To confirm that this effect was due to the secretion of Fgl2 by CD11b^+^ cells, ELISA was performed on the supernatant from this coculture. Indeed, Fgl2 was present in the supernatant of WT but not *Fgl2*^–/–^ CD11b^+^ isolated cells (Figure 7G). Given that the level of secreted Fgl2 was similar in WT and *Fcgr2b*^–/–^ coculture with WT CD11b^+^ cells, the increased cell death observed in WT but not *Fcgr2b*^–/–^ OVA-specific CD8^+^ T cells supports the conclusion that Fgl2 secreted by macrophages induces apoptosis of antigen-specific CD8^+^ T cells through CD8^+^ T cell–expressed FcγRIIB.

## Discussion

Cell-extrinsic factors driven by the formation of tumors can suppress the CD8^+^ T cell response both locally as well as systemically to limit immunotherapeutic success. While the local tumor microenvironment has been intensely studied, tumor-driving systemic perturbations are lesser known. Specifically, the elevated production of chemokines, metabolites, and growth factors (e.g., IL-6, TGF-β, VEG-F, etc.) by tumor-induced immune cells can cause systemic changes that promote suppression of adaptive immunity (53–58). Here, we show that Fgl2 is elevated in the setting of melanoma and is immunosuppressive to the T cell antitumor response. We show that this reduced response is due to immune cell–derived and not tumor-derived Fgl2 that regulates FcγRIIB^+^CD8^+^ T cells. Mechanistically, this regulation occurs in a direct manner and not through another cell type, as genetic deletion of *Fcgr2b* in a CD8^+^ T cell renders the T cell insensitive to negative regulation via Fgl2. The translational value of these studies is underscored by the expression of *Fgl2* in myeloid cells across 10 cancer types in humans. Upon further inquiry, we found that although Fgl2 can be expressed by a myriad of cell types, CD11b^+^F4/80^+^ macrophages robustly express and secrete Fgl2 in mice. After coculture of CD11b^+^ cells isolated from a tumor-challenged mice with OVA-specific CD8^+^ T cells, FcγRIIB-competent CD8^+^ T cells were selectively driven to apoptosis by WT but not *Fgl2*^–/–^ CD11b^+^ cells, a phenomenon that we did not observe in *Fcgr2b*^–/–^ OVA-specific CD8^+^ T cells. From these data, we conclude that macrophage-secreted Fgl2 negatively impacts the CD8^+^ T cell response through apoptosis of effector-like memory FcγRIIB^+^CD8^+^ T cells. Importantly, FcγRIIB is not present on naive CD8^+^ T cells, a potential reason why FcγRIIB was long thought to be absent on CD8^+^ T cells. This work suggests a model in which the presence of a tumor drives an increase in FcγRIIB on CD8^+^ T cells and in the systemic level of sFgl2. However, the increased frequency of FcγRIIB^+^CD8^+^ T cells at the tumor coupled with the increased secretion of Fgl2 by CD11b^+^ cells at the tumor further amplifies this negative feedback loop, resulting in a curtailed T cell antitumor response.

This work substantiates previous work from our laboratory demonstrating that the immunosuppressive cytokine, Fgl2, can bind FcγRIIB^+^CD8^+^ T cells in vitro (20) and provides evidence that this phenomenon occurs in vivo in the setting of cancer. The data herein are in agreement with recent work by others using the B16 model of melanoma that show that B16 cells are not a major cellular source of Fgl2 in mice (39, 43). These data also extend the work of others that have shown that administration of an antibody blocking Fgl2 has enhanced antitumor efficacy (35, 37, 38, 43, 44) and provides a CD8^+^ T cell–direct pathway that may underpin the antitumor utility of this therapy. These data weave together the discoveries from our own lab showing that FcγRIIB is expressed on CD8^+^ T cells with the discoveries of others that Fgl2 can negatively regulate FcγRIIB-expressing non-T cell types (46). Furthermore, our recently published work studies the impact of CD8^+^ T cell–derived Fgl2 in an autoregulatory axis in T cell immunity (24). The work herein describes a complementary source of Fgl2 from macrophages that regulates T cell immunity. We have previously demonstrated that FcγRIIB^+^ T cells exhibit increased proliferation (Ki67^+^), cytokine production (IFN-γ, TNF), and cytolytic ability (CD107a, GzmB) as compared with FcγRIIB^–^CD8^+^ T cells (20, 22, 23); thus, the elimination of these high-quality effector T cells is likely to hinder a productive antitumor immune response. As the myeloid compartment helps create the immunosuppressive milieu at the tumor, targeting receptor-ligand pairs to modulate the tumor environment has emerged as a promising therapeutic option. As the cell death of tumor-infiltrating lymphocytes has been shown to exacerbate T cell exhaustion in several mouse models of cancer (59, 60), targeting Fgl2 may provide additional immunotherapeutic efficacy akin to therapeutic antibodies targeting myeloid-associated receptors such as LAIR-1/2 (ClinicalTrials.gov NCT04408599) (61, 62) and Siglec-15 (ClinicalTrials.gov NCT03665285) (63, 64) currently in clinical trials, or the more established PD-L1/L2 blockade (65, 66). Additionally, rendering T cells insensitive to Fgl2 binding through FcγRIIB by engineering *Fcgr2b*^–/–^ tumor-infiltrating lymphocyte or CAR-T therapies may preserve high-quality T cells important to the antitumor response.

In sum, this work further solidifies the importance of FcγRIIB^+^CD8^+^ T cells in the antitumor response, as previously shown in our studies (22, 23). The finding that CD8^+^ T cells can be regulated in a cell-intrinsic fashion through FcγRIIB has wide implications for immunotherapies and provokes the question of what other molecules may be ligands of CD8^+^ T cell–expressed FcγRIIB. The identification of this receptor-ligand pair may be a tumor immune evasion mechanism that could be manipulated to promote T cell activity, stave off T cell exhaustion, and in turn improve patient immunotherapeutic response.

## Methods

### Sex as a biological variable.

Both male and female mice were used in the studies herein to account for sex as a biological variable and similar findings are reported for both sexes.

### Mice.

OT-I (67) and OT-II (68) transgenic mice were purchased from Taconic Farms and bred with Thy1.1^+^ (B6.PL-*Thy1^a^*/CyJ, The Jackson Laboratory, stock 000406) animals at Emory University. Pmel-17 transgenic mice possessing the TCR specific for the mouse homolog of human premelanosome protein (gp100) were also acquired from The Jackson Laboratory (B6.Cg-*Thy1^a^*/Cy Tg(TcraTcrb)8Rest/J, stock 005023) and bred with Thy1.1^+^ mice. *Fgl2*^–/–^ and Fgl2-transgenic mice were gifts of Gary Levy (26, 69) from the University of Toronto. EM:06078 Fcgr2b Fcgr2bB6null B6(Cg)-Fcgr2btm12Sjv/Cnbc (or *Fcgr2b*^–/–^) mice were obtained under an MTA from the Leiden University Medical Center and JS Verbeek. Cryopreserved embryos were shipped from the European Mutant Mouse Archive (EMMA) and re-derived at the Emory University Transgenic Mouse Core Facility. *Fcgr2b*^–/–^ mice (70) were bred with OT-I transgenic mice at Emory University. Mice were 6–10 weeks old at the start of the experiment. All experiments were performed under general anesthesia with maximal effort made to minimize suffering. All animals were housed in specific pathogen–free animal facilities at Emory University.

### Tumor cell line culture and injection.

The B16 melanoma cell line engineered to express the OVA epitope was provided by Yang-Xin Fu, University of Texas Southwest (71). B16 melanoma cells engineered to express gp100 were supplied in-house. B16-F10 cells were purchased from American Type Culture Collection (ATCC). B16-OVA and parental B16-F10 cells were cultured in RPMI 1640 (Sigma-Aldrich) supplemented with 10% FBS, 1% penicillin/streptomycin, 1% HEPES, 1% L-glutamine, and 0.05 mM 2-ME. Guidelines for B16 culture and cryopreservation outlined by ATCC were followed. Cancer cells were treated with 0.05% trypsin, washed with cold PBS, and filtered prior to cancer cell inoculation. B16-OVA, B16-gp100, or B16-F10 cells (2 × 10^5^ to 3 × 10^5^ cells) were injected in cold PBS subcutaneously into the right flank according to the protocol established by Overwijk et al. (72). Tumor volume was monitored using electronic calipers and tumor size was calculated using the following formula: tumor volume (mm^3^) = (*L* × *W*^2^)/2, where *L* is the length and *W* is the width of the tumor. Animals were sacrificed when tumors reached 2 cm in either dimension (IACUC endpoint).

### Adoptive transfers.

To monitor antigen-specific donor-reactive CD8^+^ T cell responses, 1 × 10^6^ OT-I or pmel-17 transgenic T cells were harvested from the spleens of 6- to 10-week-old mice, filtered through 70- and 40-μm filters, and adoptively transferred through intravenous injection into naive mice 24 hours before tumor injection. For co-adoptive transfer experiments, 5 × 10^5^ WT and 5 × 10^5^ *Fcgr2b*^–/–^ OT-I were given at a 1:1 ratio, confirmed by flow cytometry the day before naive mice were challenged with tumors. In all adoptive transfers, cells were counted using a Nexcelom Cellometer (Nexcelom Bioscience) and stained with CD8-BUV805, CD4-BUV496, Thy1.1-BV711, Thy1.2-PerCP, Vα2-FITC, and Vβ5-PE or Vβ13-PE antibodies (BioLegend). Antibody details are provided in Supplemental Table 3. Frequency of OT-I cells was determined via CD8^+^Thy.1.1^+^ Vα2 and Vβ5 TCR coexpression. Frequency of pmel-17 cells was determined via CD8^+^Thy.1.1^+^ Vα2 and Vβ13 TCR coexpression.

### Tumor lysate.

Tumors from WT or *Fgl2*^–/–^ mice were harvested and processed through 70- and 40-μm filters with cold PBS prior to resuspending cells in cold lysis buffer at a concentration of 1 × 10^7^ cells/mL. After agitation, samples were centrifuged to remove cellular debris. The eluent was stored at –20°C until use in Fgl2 ELISA.

### Bone marrow chimera generation.

To generate *Fgl2*^–/–^ bone marrow chimeras, CD45.1^+^ Pepboys (Charles River Laboratories) were irradiated with 4.5 Gy radiation 2 times, 6 hours apart. Bone marrow was collected from the tibias and femurs of donor CD45.2^+^ WT or *Fgl2*^–/–^ mice. Irradiated male mice were given 20 million donor cells through intravenous injection. Water containing polymyxin B (Sigma-Aldrich) and neomycin (Sigma-Aldrich) was administered to these mice for 8 weeks after irradiation. After 6 and 8 weeks, engraftment and chimerism was confirmed by cheek bleed for the frequency of CD45.2^+^ transferred cells via flow cytometry.

### Human samples.

For measurement of Fgl2 in human serum, blood samples were drawn from healthy donors or from patients undergoing treatment at Emory University Hospital for melanoma between 2009 and 2019. Serum was isolated and analyzed using the LEGEND MAX Human Fgl2 ELISA kit (BioLegend).

### Demographic information.

Demographic data for healthy donors can be found in Supplemental Table 1 and demographic data for patients with melanoma can be found in Supplemental Table 2. A limitation of our study is the inability to generalize findings across racially diverse patient populations, as our patient population with melanoma did not include any non-White patients.

### Mouse Fgl2 ELISA.

For detection of Fgl2 in mice, blood was taken from mice via cheek bleed prior to B16 challenge and 10 days after B16 challenge. Serum was isolated using BD Microtainer Tubes and Fgl2 protein levels were detected with Fgl2 ELISA (Aviva Systems Biology). To quantify secreted Fgl2 in the cell supernatant, the Mouse FGL2/Prothrombinase ELISA kit (Abcam) was used.

### Murine cell processing and flow cytometry staining.

Spleen, draining lymph node (right inguinal proximal to tumor), and tumors of mice were processed to cell suspensions with 70- and 40-μm filters and blood underwent red blood cell (RBC) lysis prior to staining. Samples were stained for surface markers using the antibodies listed in Supplemental Table 3. For assessment of cell death, cells were harvested and caspase 3/7 and 7-AAD staining was performed with the CellEvent Caspase-3/7 Green Flow Cytometry Assay kit (Thermo Fisher Scientific). For intracellular cytokine staining, B16-OVA tumors of WT and *Fgl2*^–/–^ mice were homogenized and cells were ex vivo stimulated for 4 hours with 10 nM OVA_257–264_ (SIINFEKL) peptide and 10 μg/mL GolgiPlug (BD Biosciences). For baseline studies, the spleens of healthy mice were homogenized and splenocytes were stimulated with 30 ng/mL PMA, 400 ng/mL ionomycin, and 10 μg/mL GolgiPlug. After 4 hours, cells were processed, fixed, and stained for flow cytometry analyses. Samples without peptide or PMA/ionomycin were analyzed for unstimulated controls. All flow cytometry samples were acquired on a Fortessa flow cytometer (BD Biosciences) and data were analyzed using FlowJo (v10) and Prism 10 (GraphPad Software). Absolute cell numbers were calculated using CountBright Beads (Life Technologies) according to the manufacturer’s instructions.

### Reanalysis of publicly available RNA-sequencing datasets.

To begin to determine the significance of Fgl2 expression in melanoma patients, the TIMER2.0 database (48–50) was used to visualize the correlation between Fgl2 expression and patients with primary versus metastatic SKCM. The EPIC algorithm was employed to show the correlation between *Fgl2* expression (log_2_ transcripts per million) and macrophage tumor infiltration level in patients with primary melanoma (*n* = 103) and metastatic melanoma (*n* = 368). The ρ and *P* values were generated in the TIMER database. To further investigate the expression of *Fgl2* in patients with cancer, 2 additional independent RNA-sequencing datasets were reanalyzed with the GEO2R platform and the pipeline established in BBrowser2 (BioTuring) (73). Combes et al. (51) performed bulk RNA sequencing of tumor-infiltrating cells containing live cells, stroma, tumor cells, Tconv, Treg, and myeloid cells from 12 distinct solid tumor types. For our analyses, cancer types with fewer than 3 samples in any of the tumor-infiltrating populations were omitted. In the second dataset deposited by Sade Feldman et al. (52), single-cell RNA sequencing of 16,291 cells from 32 patients with melanoma was performed using SMART-Seq2. We next investigated *Fgl2* expression in 6 syngeneic mouse tumor models. In the single-cell RNA sequencing dataset deposited by Kumar et al. (74), the tumor was dissociated and CD45^+^ cells were sorted prior to sequencing. Data plotting and statistical analysis were performed with GraphPad Prism 10. Datasets are available in the NCBI GEO database under accession numbers GSE184398, GSE120575, and GSE121861.

### CD11b^+^ cell isolation and OT-I coculture, Fgl2 ELISA of cell supernatant.

Fourteen days after WT or *Fgl2*^–/–^ mice were challenged with B16, spleens and tumors were harvested and CD11b^+^ splenocytes were enriched with MACS enrichment using CD11b microbeads (Miltenyi Biotec). Naive spleens were used as well. Purity was determined by flow cytometry and cell number was determined using a Nexcelom Cellometer (Nexcelom Bioscience). Twenty-four hours later, the supernatant was collected for Fgl2 ELISA. For OT-I coculture experiments, WT or *Fcgr2b*^–/–^ OT-I CD8^+^ T cells were stimulated 2 days prior to CD11b^+^ cell isolation with 10 nM SIINFEKL peptide, 10 ng/ml IL-2, and 1 μg/mL of anti-CD28 antibody (clone 37.51, Thermo Fisher Scientific) in RPMI 1640 (Sigma-Aldrich) and supplemented with 10% FBS, 1% penicillin/streptomycin, 1% HEPES, 1% L-glutamine, and 0.05 mM 2-ME. Upon CD11b^+^ isolation as described above, 2-day-stimulated OT-I T cells were resuspended in fresh media with peptide and WT or *Fgl2*^–/–^ CD11b^+^ cells at a 1:1 ratio. Twenty-four hours later, cell supernatant was collected for Fgl2 ELISA and cells were collected for caspase 3/7 and 7AAD staining using the CellEvent Caspase-3/7 Green Flow Cytometry Assay Kit (Thermo Fisher Scientific).

### Statistics.

The Mann-Whitney nonparametric, unpaired *t* test was used to compare cell populations between groups, while Wilcoxon’s matched-pairs rank tests were performed to compare subsets within the same donor. Kruskall-Wallis 1-way ANOVA with multiple comparisons was performed when comparing multiple groups. The ROUT outliers test was used to determine any outliers. All analyses were done using Prism 10 (GraphPad Software). In all legends and figures, mean + SEM are shown. A *P* value of less than 0.05 was considered significant.

### Study approval.

This study was carried out in strict accordance with the recommendations in the NIH *Guide for the Care and Use of Laboratory Animals* (National Academies Press, 2011). The protocol (PROTO201700558) was approved by the Institutional Animal Care and Use Committee of Emory University. The protocol for obtaining patient serum was approved by Emory University’s Institutional Review Board (IRB no. 00046593). Written consent was obtained prior to participation in human studies.

### Data availability.

All raw data files reported in this manuscript are contained in the Supporting Data Values file. All genomic/transcriptomic data used in this study are available in the GEO database under accession numbers GSE184398, GSE120575, and GSE121861.

## Author contributions

KBB designed, performed, and analyzed experiments and wrote the manuscript. JMRB, DL, and MW performed experiments. CMP provided the gp100-expressing B16 melanoma cells, technical expertise, and editing of the manuscript. MLF designed experiments, provided funding, and edited the manuscript.

## Supplementary Material

Supplemental data

Supporting data values

## Version 1 03/24/2025

Electronic publication
