# Supplementary material for: Macrophage-derived Fgl2 dampens antitumor immunity through regulation of FcγRIIB+CD8+ T cells in melanoma
Source: JCI Insight. 2025 Mar 24;10(6):e182563. doi: 10.1172/jci.insight.182563 (PMC11949062; doi:10.1172/jci.insight.182563)
Supplement: Supplemental data [file jciinsight-10-182563-s105.pdf]

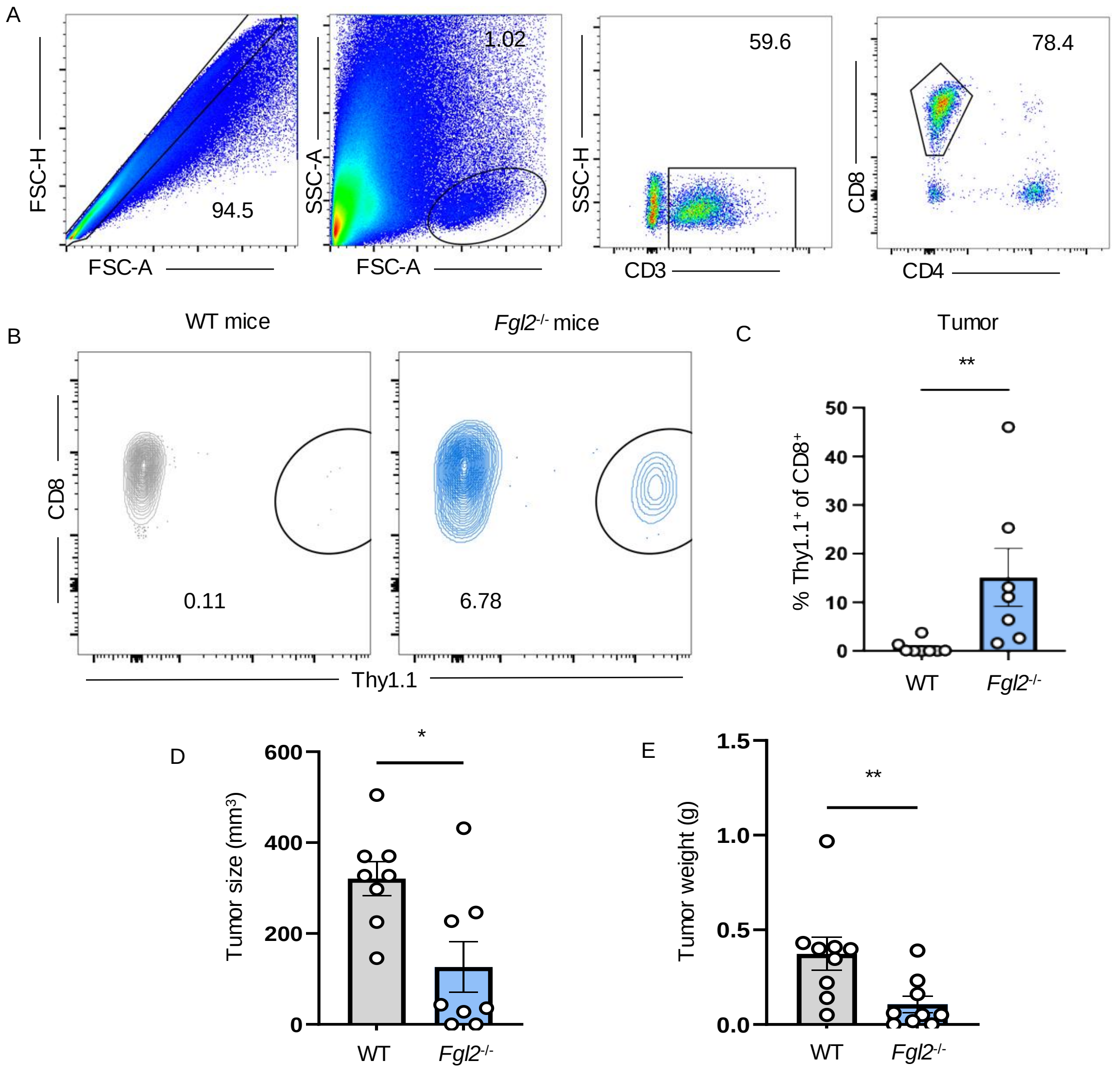

Supplemental Figure 1. Fgl2 is immunosuppressive to tumor-specific CD8⁺ T cells in a gp100/pmel-17 B16 model of melanoma. (A) Gating strategy, (B) representative flow, and (C) summary data showing frequency of pmel-17 CD8⁺ (Thy1.1⁺) at the tumor, (D) tumor size, and (E) tumor weight in WT vs *Fgl2⁻/⁻* mice 14 days post tumor challenge (n=7-9, pooled data from two experiments). Mann-Whitney non-parametric, unpaired test was used when comparing two groups. The error bar in summary figures denotes mean ± SEM. *p<0.05 **p<0.01.

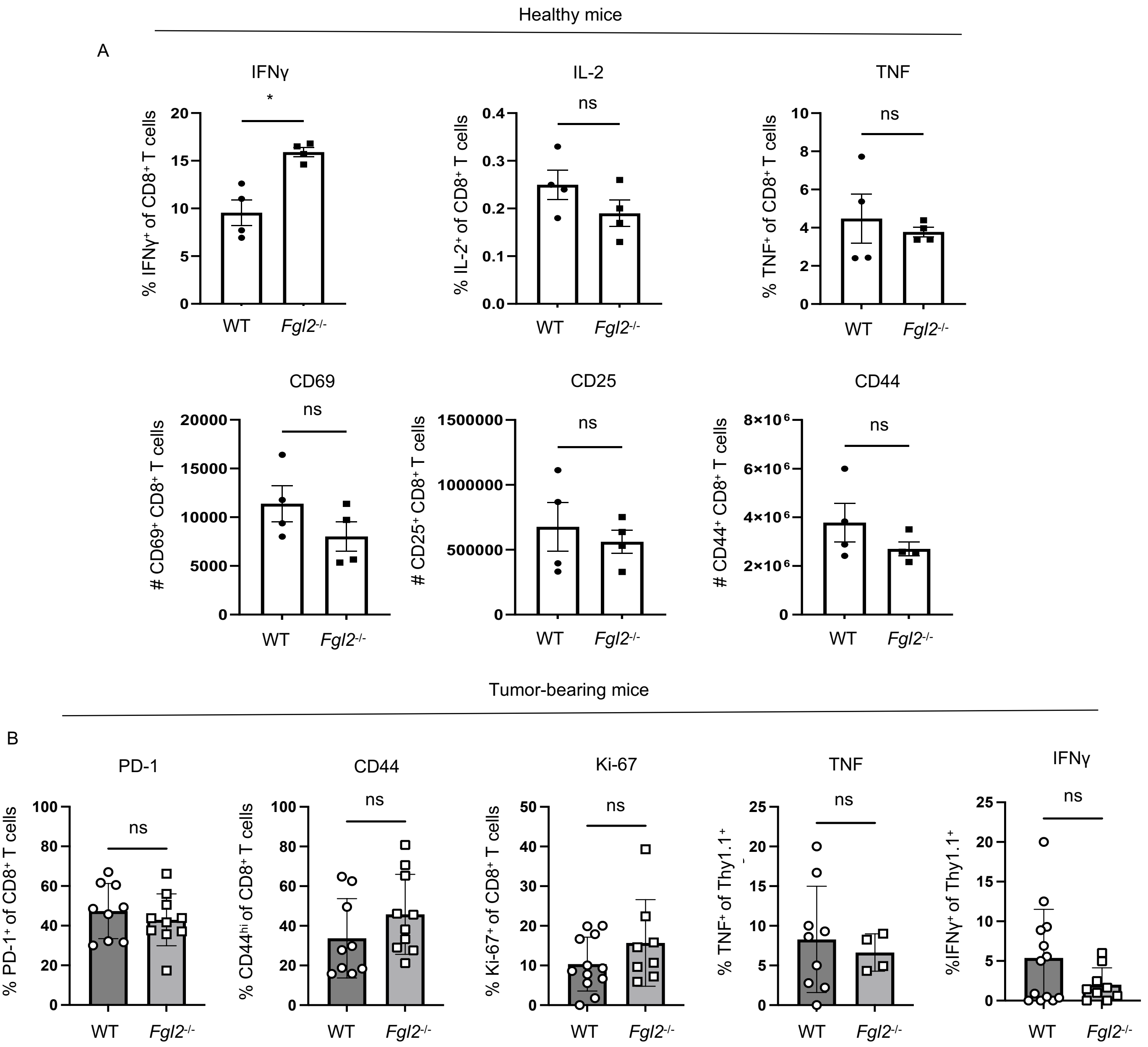

Supplemental Figure 2. Profiling of CD8⁺ T cells from WT and Fgl2-deficient healthy and tumor-bearing mice. (A) Summary data showing frequency of IFNγ⁺, IL-2⁺, TNF⁺, CD69⁺, CD25⁺, and CD44⁺ among CD8⁺ T cells from the spleen of healthy WT or *Fgl2-/-* mice (n=4). (B) Summary data showing frequency of PD-1⁺, CD44⁺, Ki-67⁺ among CD8⁺ T cells and TNF⁺, and IFNγ⁺ among OT-I (Thy1.1⁺) from the tumor of B16-OVA tumor bearing WT or *Fgl2-/-* mice (n=4-10, pooled data from two experiments). Mann-Whitney non-parametric, unpaired test was used when comparing two groups. The error bar in summary figures denotes mean ± SEM. Ns=not significant, *p<0.05.

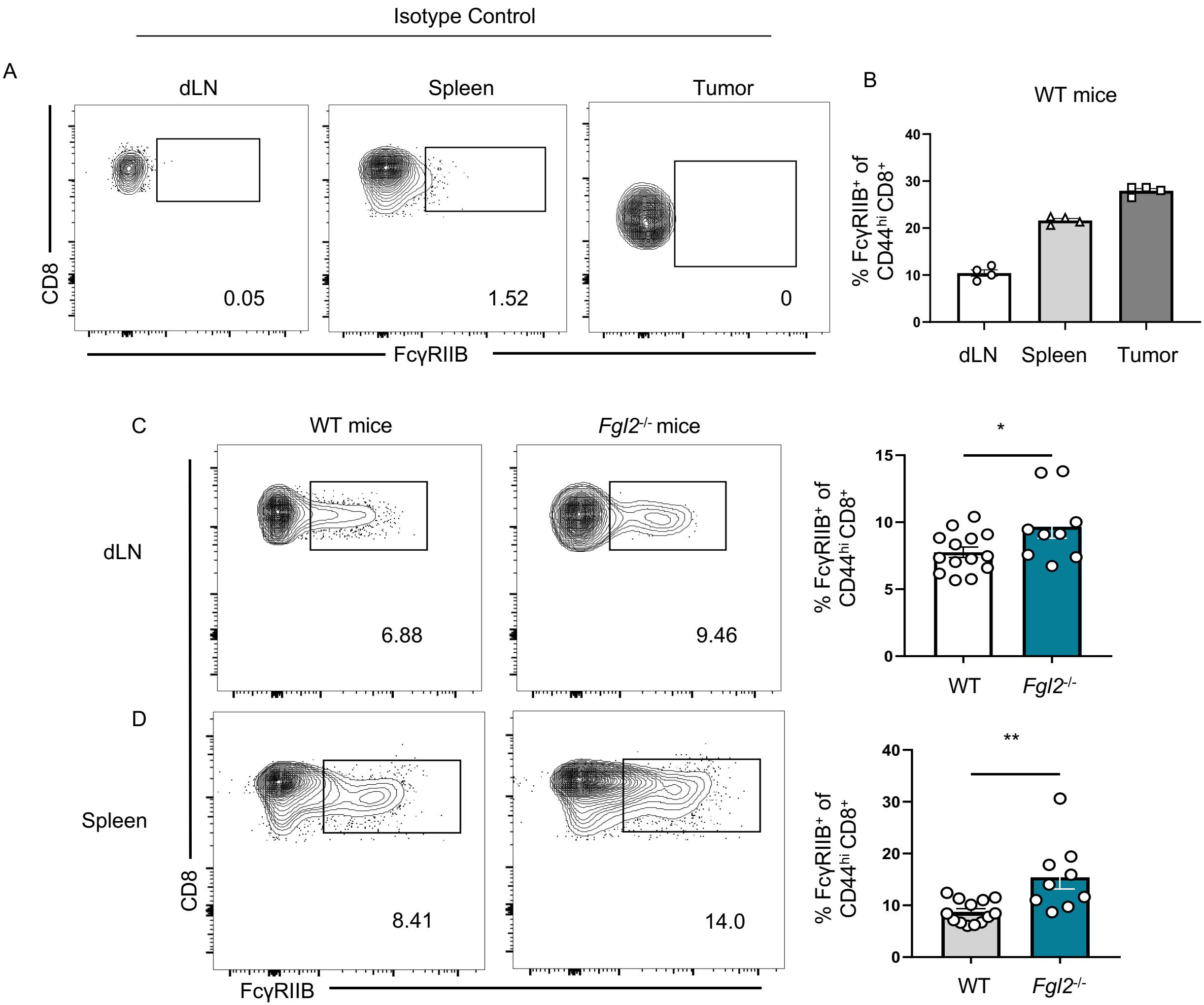

Supplemental Figure 3. Host Fgl2 negatively regulates FcγRIIB⁺ CD8⁺ T cells at the lymph nodes and spleen of mice. (A) Representative flow cytometry plots showing isotype control staining. (B) Summary data showing frequency of FcγRIIB⁺ among CD44^{hi} CD8⁺ T cells in the dLN, spleen, and tumor of WT B16-challenged mice (n=4). Representative flow and summary data showing the frequency of FcγRIIB⁺ among CD44^{hi} CD8⁺ T cells in the (C) dLN and (D) spleen of WT vs *Fgl2*$^{-/-}$ B16-challenged mice (n=9-14, pooled data from two experiments). Mann-Whitney non-parametric, unpaired test was used when comparing two groups. The error bar in summary figures denotes mean ± SEM. *p<0.05 **p<0.01.

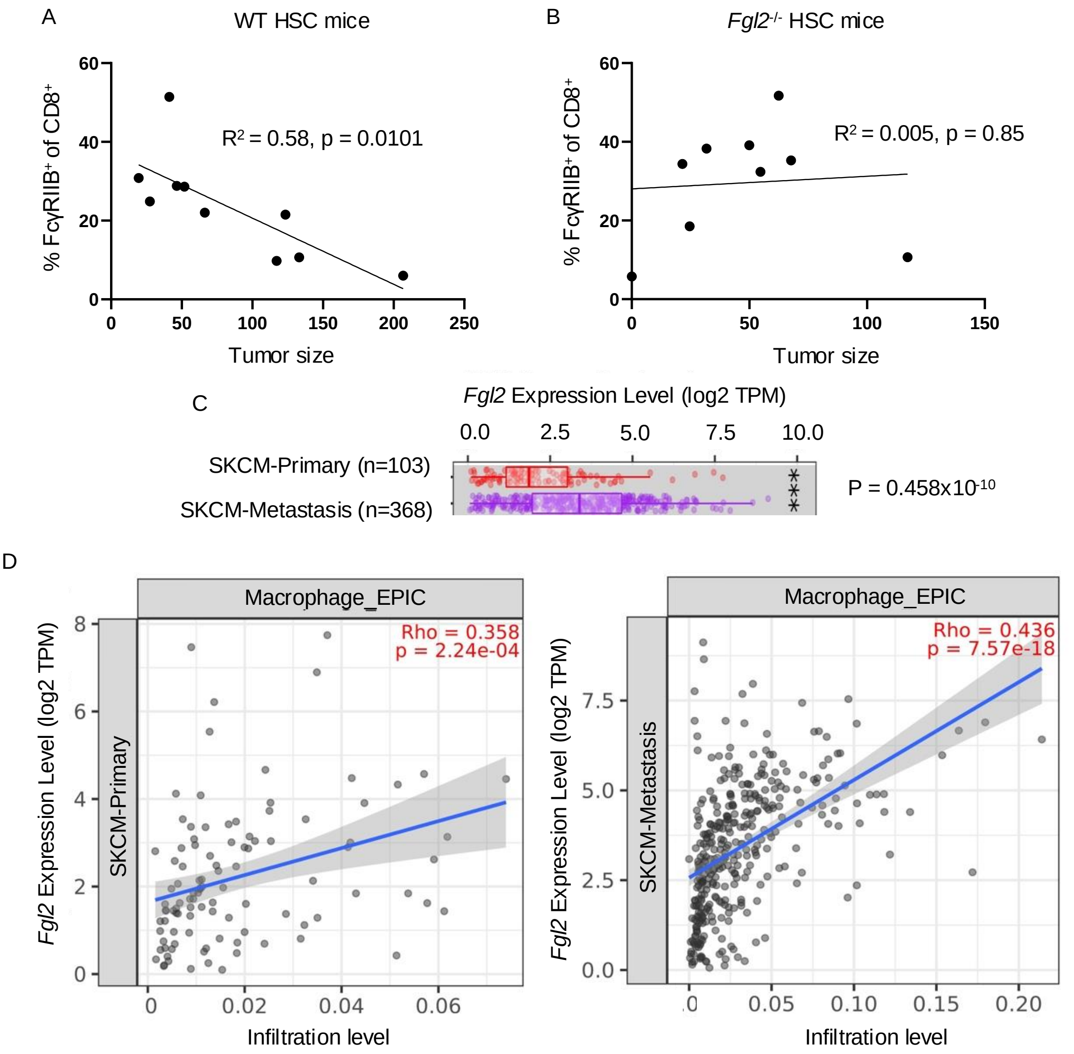

Supplemental Figure 4. Fgl2 is correlated with tumor progression in melanoma-bearing mice and patients and is associated with macrophage tumor infiltration in SKCM. (A) Linear regression showing the correlation between the frequency of FcγRIIB+ CD8+ T cells in mice reconstituted with (A) WT hematopoietic stem cells (HSC) vs. (B) Fgl2-/- HSC and B16 tumor size during tumor challenge. (C) Dot plot showing Fgl2 expression level (log2 TPM) datamined from the TIMER database at the primary tumor of patients with SKCM (SKCM-primary) (n=103) vs patients with melanoma metastasis (SKCM-metastasis) (n=368). (D) Linear regressions relating Fgl2 expression level (log2 TPM) in SKCM primary tumor (n=103) and SKCM metastasis (n=368) with estimated infiltration level of macrophages according to the EPIC algorithm. The R^2, rho correlation coefficient, and p-value is shown in each corresponding graph.

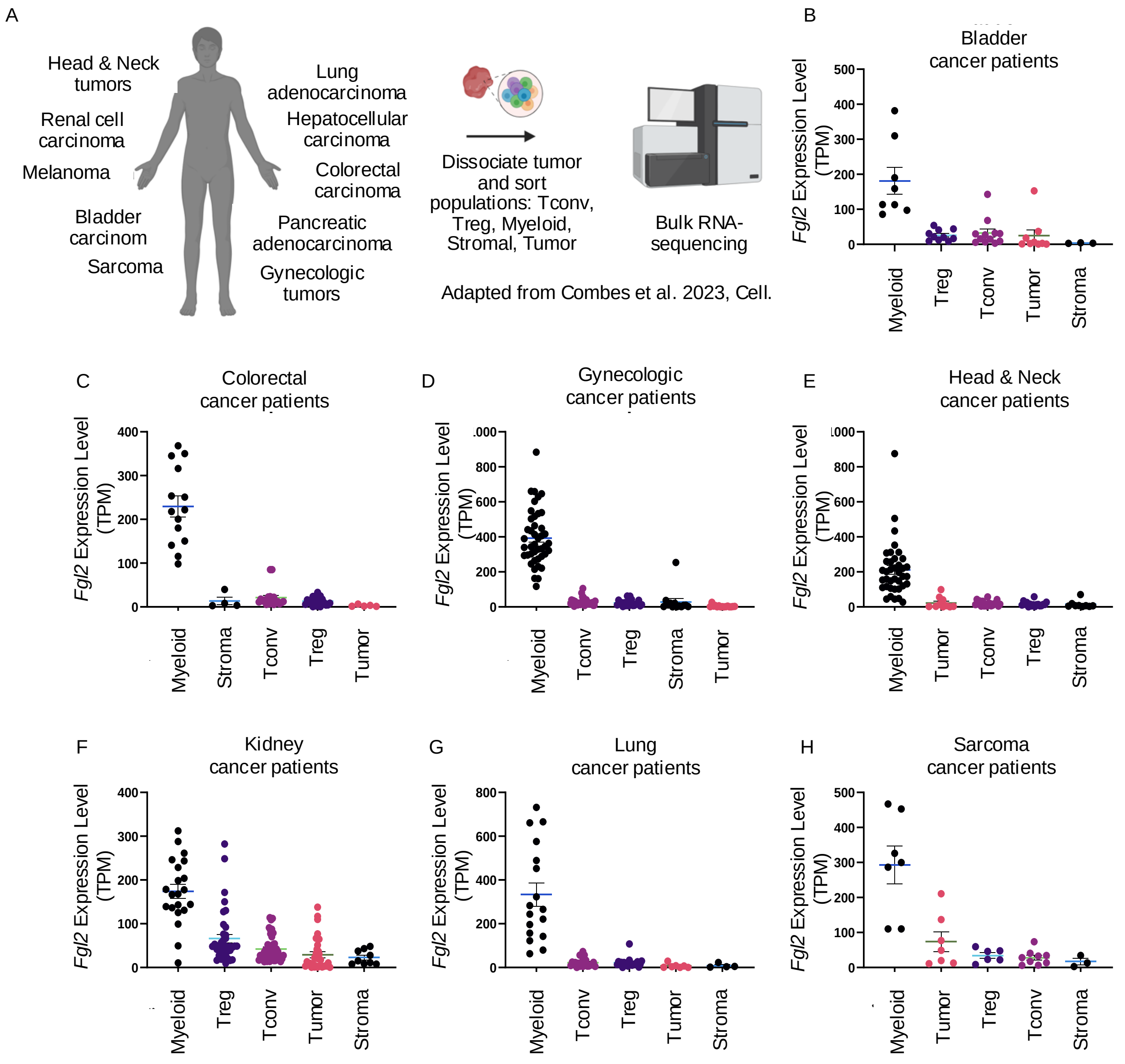

Supplemental Figure 5. Myeloid cells are a major cellular source of Fgl2 in humans across cancer types. (A) Schematic of the workflow previously published by Combes et al. showing dissociation of tumors for bulk RNA sequencing from patients with head and neck tumors (n=38), lung adenocarcinoma (n=17), renal cell carcinoma (n=21), hepatocellular carcinoma (n=3), melanoma (n=7), colorectal carcinoma (n=14), bladder carcinoma (n=8), pancreatic adenocarcinoma (n=4), gynecologic tumors (n=43), and sarcomas (n=7). Cancer types were excluded if less than three samples were present in the myeloid population in the cancer type (B-H) Dot plots showing the expression of Fgl2 in the sorted myeloid, stroma, Treg, Tconv, and tumor populations in patients across seven cancer types.
